# Brain Abnormalities and Glioma-Like Lesions in Mice Overexpressing the Long Isoform of PDGF-A in Astrocytic Cells

**DOI:** 10.1371/journal.pone.0018303

**Published:** 2011-04-07

**Authors:** Inga Nazarenko, Anna Hedrén, Hanna Sjödin, Abiel Orrego, Johanna Andrae, Gijs B. Afink, Monica Nistér, Mikael S. Lindström

**Affiliations:** 1 Department of Oncology-Pathology, Karolinska Institutet, Stockholm, Sweden; 2 University of Pittsburgh, Pittsburgh, Pennsylvania, United States of America; 3 Department of Medical Biochemistry and Biophysics, Karolinska Institutet, Stockholm, Sweden; 4 Laboratory for Reproductive Biology, Academic Medical Center, University of Amsterdam, Amsterdam, The Netherlands; 5 Ludwig Institute for Cancer Research, Stockholm Branch, Karolinska Institutet, Stockholm, Sweden; University of California, Los Angeles, and Cedars-Sinai Medical Center, United States of America

## Abstract

**Background:**

Deregulation of platelet-derived growth factor (PDGF) signaling is a hallmark of malignant glioma. Two alternatively spliced PDGF-A mRNAs have been described, corresponding to a long (L) and a short (S) isoform of PDGF-A. In contrast to PDGF-A(S), the PDGF-A(L) isoform has a lysine and arginine rich carboxy-terminal extension that acts as an extracellular matrix retention motif. However, the exact role of PDGF-A(L) and how it functionally differs from the shorter isoform is not well understood.

**Methodology/Principal Findings:**

We overexpressed PDGF-A(L) as a transgene under control of the glial fibrillary acidic protein (GFAP) promoter in the mouse brain. This directs expression of the transgene to astrocytic cells and GFAP expressing neural stem cells throughout the developing and adult central nervous system. Transgenic mice exhibited a phenotype with enlarged skull at approximately 6-16 weeks of age and they died between 1.5 months and 2 years of age. We detected an increased number of undifferentiated cells in all areas of transgene expression, such as in the subependymal zone around the lateral ventricle and in the cerebellar medulla. The cells stained positive for Pdgfr-α, Olig2 and NG2 but this population did only partially overlap with cells positive for Gfap and the transgene reporter. Interestingly, a few mice presented with overt neoplastic glioma-like lesions composed of both Olig2 and Gfap positive cell populations and with microvascular proliferation, in a wild-type p53 background.

**Conclusions:**

Our findings show that PDGF-A(L) can induce accumulation of immature cells in the mouse brain. The strong expression of NG2, Pdgfr-α and Olig2 in PDGF-A(L) brains suggests that a fraction of these cells are oligodendrocyte progenitors. In addition, accumulation of fluid in the subarachnoid space and skull enlargement indicate that an increased intracranial pressure contributed to the observed lethality.

## Introduction

The family of platelet-derived growth factor (PDGF) includes four different polypeptides: PDGF-A, -B, -C and -D, encoded by four different genes [1]. Difference in structure and proteolytic processing places these ligands into two subfamilies: PDGF-A and -B constitutes one subfamily, and PDGF-C and –D a second subfamily [2]. PDGF-A affects cell proliferation, survival, migration and differentiation by way of paracrine or autocrine interaction with the cell surface protein tyrosine kinase receptor PDGFR-α [1], [2], [3]. The gene organization of *PDGFA* and *PDGFB* includes seven exons. Exon number 6 in both *PDGFA* and *–B* encodes a highly basic stretch of 18 amino acids with a high proportion of lysine and arginine at the carboxy-terminus. This positively charged retention motif can associate with heparin/heparan sulfate proteoglycans of the extracellular matrix [4]. PDGF-A exists as two isoforms corresponding to a long (L) and a short (S) form due to alternative splicing of exon 6 [5]. Consequently, PDGF-A_L_ has a basic carboxy-terminal tail encoded by exon 6, attaching it to the extracellular matrix whereas PDGF-A_S_ is freely diffusible in the extracellular fluid since it lacks this retention motif [3], [6].

The role of the basic extension in PDGF-A_L_ and how it makes the long form functionally different from the short remain unknown, though the significance of this motif is indicated by the fact that it is highly similar to a corresponding sequence in PDGF-B. A similar structural motif has also been found in placental growth factor (PlGF) [7] and in the longer splice variants of vascular endothelial growth factor (VEGF) [8], which are potent mitogens for endothelial cells and structurally related to PDGF. Importantly, the basic stretch of amino acids in PDGF-A_L_ is evolutionary conserved between frog, mouse and man [9], [10]. Expression of the long form of PDGF-A was originally identified in tumor cells [5], [11], [12], and PDGF-A_L_ was cloned from a human glioma cell line [11]. The presence of mRNA encoding the long PDGF-A form in tumor cells led to the suggestion that it might be an important component of the transformed phenotype in some cells. By now it is known that the short and long forms of PDGF-A transcripts are expressed in a variety of normal tissues, such as liver, kidney and thymus, although the short form of PDGF-A is predominant in heart, muscle and brain [13], [14]. Interestingly, PDGF-A_L_ appears not to be present in the retina [15] or spinal cord [16].

A number of genetically modified mouse lines with gain- and loss-of-function mutations in *Pdgf* and *Pdgf* receptor genes have been created in order to understand PDGFs physiological functions. Phenotype analyses of knockout mice have demonstrated the important role of PDGFs and PDGF receptors during embryonic development [17]. The *Pdgfa* knockout is invariably lethal, but displays a range of time points of lethality, spanning from embryonal day (E)10 to postnatal day (P)60 [17], [18]. Further analysis of *Pdgfa* null mice supported the findings from *in vitro* studies, suggesting a role for PDGF-A as a mitogen for oligodendrocyte progenitors [19], [20], [21]. Postnatally surviving *Pdgfa* knockout mice develop tremor due to severe hypomyelination of the neuronal projections within the CNS [22], while overexpression of PDGF-A_S_ in the developing CNS instead leads to hyperproliferation of oligodendrocyte progenitors (OPs) [16].

Even though the importance of PDGF-A and its receptor PDGFR-α has been well described in the above mentioned studies, these investigations did not distinguish between the individual PDGF-A isoforms. To further investigate the function of the long form of PDGF-A *in vivo*, we have derived a transgenic mouse model overexpressing PDGF-A_L_ under control of the human glial fibrillary acidic protein (hGFAP) promoter. This promoter is active in mouse embryonic brain already from day E9.5 in the telencephalon and cerebellar region, with an activity peak around birth. The hGFAP promoter remains active throughout mouse adult life in astrocytic cells of the entire brain and particularly in astrocytes of the glia limitans and in Bergman glia of the cerebellum [23]. Neural stem cells in the subventricular zone of the forebrain and in the subgranular zone of the dentate gyrus in hippocampus display astrocytic characteristics by Gfap expression and Gfap promoter activity [24], [25], [26], [27]. Therefore, by using this promoter, we are introducing PDGF-A_L_ also into the neural stem cell niches of developing and adult mouse brains. We found that PDGF-A_L_ similarly to the short form can induce abnormal accumulation of cells in the mouse brain. However, the increased cell numbers were not adjusted with time, leading to glioma-like lesions in 4 out of 22 histologically analyzed brains and to lethality. We also observed accumulation of fluid in the subarachnoid space and enlargement of the skull indicating an increased intracranial pressure contributing to early death.

## Materials and Methods

### Generation of transgenic mice

**Figure 1:**
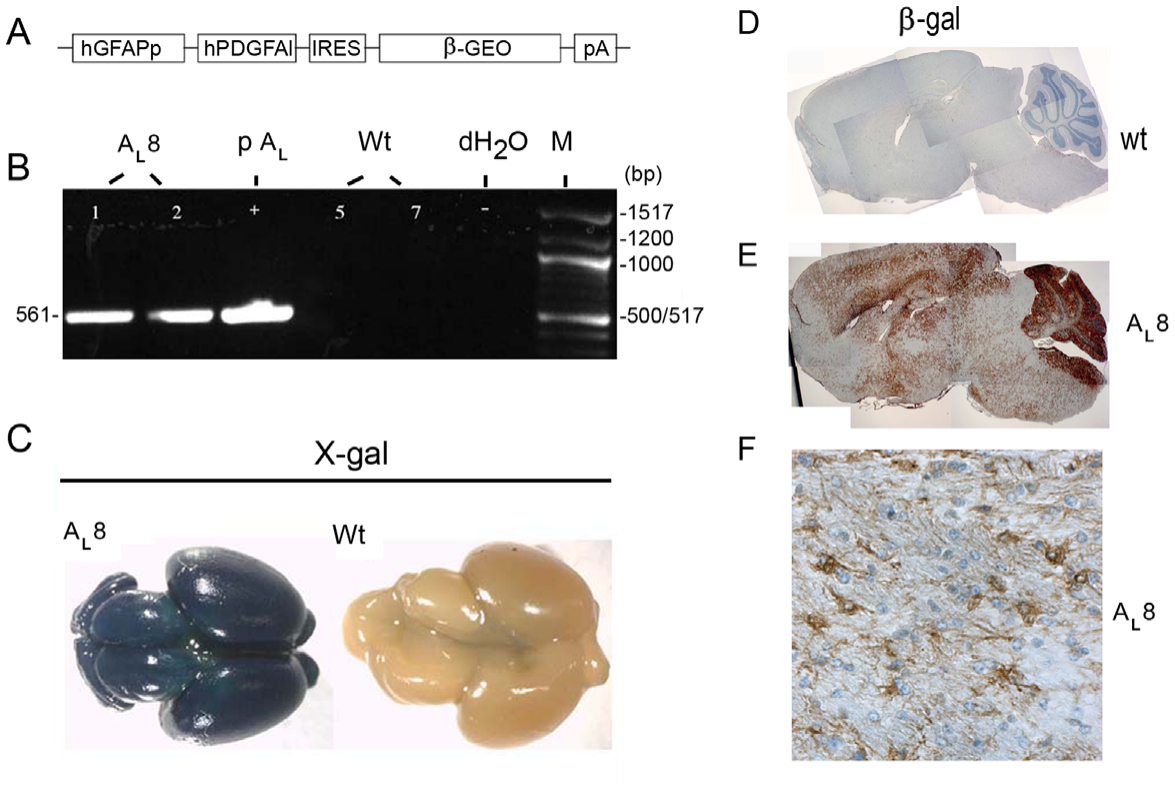
Detection and validation of transgene expression. (A) The hGFAPp-PDGFA_L_-IRES-βGeo construct as depicted in a schematic drawing. (Abbreviations: hGFAPp, human glial fibrillary acidic protein promoter; hPDGFAl, human platelet-derived growth factor A long; IRES, internal ribosome entry site; βGEO, beta-galactosidase/neomycine fusion; pA, polyadenylation signal.) (B) Results from PCR using genomic DNA from mouse tail biopsies (transgenic line, A_L_8) and primers for human PDGF-A_L_, resulting in a 561 base pair fragment. PCR primers unique for PDGF-A_L_ are situated in a way that the reverse primer corresponds to a sequence in exon 6, which is only present in PDGF-A_L_, not -A_S_. Controls are the hGFAPp-PDGFA_L_-IRES-βGEO construct (pA_L_) and DNA from wild type (wt) tails. (C) Whole mount X-gal staining of P0 transgenic and wt brains reveals activity of the transgene on the brain surface (blue color). (D–F) Presence of the β-galactosidase (β-gal) reporter in the transgenic mouse brain compared to a wt brain as visualized by IHC (brown color). A higher magnification image (F) reveals the individual β-gal+ cells, here exemplified by cells in a section from the temporal lobe of a transgenic mouse brain (40x obj.).

Transgenic mice were generated essentially in the same way as described previously [28]. A 1.8 kb human GFAP 5′ promoter fragment [23] was used to overexpress human PDGF-A_L_ (GI197333758) in the mouse brain (Figure 1). The IRESβGEO vector [29] was a backbone for the hGFAPp-PDGFA_L_-IRESβGEO construct. The plasmid IRESβGEO consists of an internal ribosome entry site, followed by a lacZ-neo^R^ (βgeo) fusion cDNA and polyadenylation signal, and was modified by introduction of a unique AscI site at the 3′ end of the polyadenylation signal. The hGFAP promoter - PDGF-A_L_ cDNA fragment with a 5′ AscI site was prepared and inserted at the unique NotI site in the modified IRESβGEO. All the steps in design of the construct IRES-βGEO (hGFAPp,PDGF-A_L_) were verified by enzyme cleavage and sequencing. Functional testing was performed *in vitro* by plasmid transfection followed by PDGF-A_L_ and β-galacatosidase (β-gal) protein detection (data not shown). Transgenic mice were generated by standard oocyte injection of the fragment excised with AscI, isolated by agarose gel electrophoresis and purified over Qiagen columns (Qiagen, Valencia, CA). The purified DNA was diluted to a final concentration of 2.0 µg/ml in microinjection buffer (10 mM Tris-HCl pH 7.3, 0.1 mM EDTA), and injected into the male pronucleus of fertilized eggs from F1 (B6CBA) x F1 (B6CBA) mice (Bommice, Denmark) at the Karolinska Center for Transgene Technologies (KCTT). Transgenic founders were identified by PCR amplification of the unique hPDGF-A_L_ sequence in tail biopsies. DNA was extracted from tail biopsies of three week old mice in lysis buffer (10 mM Tris-HCl, 5 mM EDTA, 0.2% SDS, 200 mM NaCl) containing 100 µg/ml proteinase K, and incubated at 55°C over night. The following primers for unique hPDGF-A_L_ were used: 5′ GAT ACC TCG CCC ATG TTC TG 3′ (forward) and 5′ CTG ACT CCC TAG GCC TTC C 3′ (reverse) resulting in a product with the size of 561 bp. The fragment was amplified by 10 cycles of the following incubations: 95°C for 1 minute, 60°C for 3 minutes and 72°C for 2 minutes, followed by another 30 cycles of incubations: 95°C for 1 minute, 60°C for 1 minute, and 72°C for 2 minutes. The product was visualized by electrophoresis in 0.8% agarose.

### Detection of transgene expression

Expression of transgenic protein was detected in brains from newborn mice using X-gal staining to detect activity of the β-gal reporter. Brains were dissected from mice (sacrificed using CO_2_), washed in PBS, fixed in 2% paraformaldehyde (PFA) in PBS at room temperature for one hour, permeabilized in (2 mM MgCl_2_, 0.02% NP40, 0.01% Na-deoxycholate in PBS), and stained in (2 mM MgCl_2_, 0.02% NP40, 0.01% Na-deoxycholate, 5 mM K_4_Fe(CN)_6_, 5 mM K_3_Fe(CN)_6_ in PBS with 1 mg/ml X-gal) at 37°C for a few hours. Stained brains were washed in PBS, dehydrated twice in methanol, and cleared in BABB (benzyl alcohol/benzyl benzoate 1∶2) for photography [30]. Transgene activity was also studied using immunohistochemistry (IHC) for β-gal.

### Ethics statement

All animal experiments have been approved by the local ethical committee for animal experimentation (Stockholms Norra Djurförsöksetiska Nämnd) under the ethical approvals N85/02, N81/05 and N110/08.

### Survival analysis

The mice were kept in a mixed CBA/C57/Bl6 background and were followed for up to 18 months and carefully monitored. Mice that survived for more than 6 weeks were used for breeding and all crossings were kept hemizygous. Non-transgenic littermates were used as controls. Mice were sacrificed upon signs of obvious physical changes in the shape of their skull or changes in behavior and motor functions. All mice were further subjected to necropsy and all major organs inspected and collected for histology if abnormal in any way. Brains were collected for histological analysis but unfortunately not every brain was suitable for histological analysis, as some of the mice were found dead unexpectedly in the beginning of the study. Among 26 collected PDGF-A_L_ transgenic mouse brains, 4 were not suitable for histological analysis due to severe post mortem effects. Brains were analyzed and rated according to the extent of abnormal cell accumulation using personal grading by the authors as follows: “-” - no extra cells; “+” – low number; “++” – high; “+++” – very high (Table S1). Statistica program (StatSoft) was used to compare survival of the transgenic and wild type mice in the Kaplan-Meier diagram. The *t-*test for equality of means was used to calculate the statistical significance of any mean litter size and sex ratio changes.

### Immunohistochemistry

Brain tissues from transgenic and wild type (wt) control mice were dissected and fixed in 4% PFA over night, transferred to 70% ethanol, dehydrated and paraffin embedded. They were then sectioned at 5 µm and stained with Hematoxylin-Eosin or used for IHC. We regularly used automated stainings in Ventana Discovery Automated Stainer following vendor's suggestions (Ventana Medical Systems, Tucson, Arizona). Briefly, deparaffinization was done in the Ventana machine and heat-induced epitope retrieval in Tris-Borate EDTA buffer pH 8.0 was used for the antigens. A streptavidin-biotin horseradish peroxidase based DAB kit provided by Ventana was used for detection of single antibody stainings. Slides were subjected to graded ethanol rinses, cleared in Xylene and mounted in Pertex. For double stainings of β-gal and different progenitor cell markers as well in the case of co-staining Gfap and Olig2, the sections were deparaffinized and subjected to heat-induced epitope retrieval as above. Antigens were sequentially detected, first with a streptavidin-biotin horseradish peroxidase based DAB kit and second using a streptavidin-alkaline phosphatase based kit employing NBT/BCIP (BlueMapKit). Slides were in some cases counterstained with Nuclear Fast Red as indicated in the figure.

### Analysis and documentation of immunohistochemistry results

The IHC stainings were analyzed microscopically and digital photographs were obtained using an Olympus BH2 Microscope equipped with a Leica DF320 camera. Images were assembled in Adobe Photoshop. Comparisons of the numbers of brain cells stained positive for Pdgfr-α, Sox2, Olig2, Gfap and Ki67 in the different genotypes were done by counting the numbers of stained cells in each anatomical region of interest and using at least three brains per genotype. Due to differences in cell density between wt and transgenic mice the numbers of immunoreactive cells were usually expressed as a fraction (in percent) of all cells in the selected area(s). When applicable, the t-test for equality of means was used to calculate the statistical significance of differences in mean cell numbers. A p-value of <0.05 was considered to reflect a significant difference.

### Antibodies

Primary antibodies were rabbit anti-bacterial β-gal (ab616) (Abcam) (1∶500), rabbit anti-cow GFAP (DAKO) (1∶500), rabbit anti-mouse NG2 (Millipore) (1∶500), rabbit anti-mouse Olig2 (AB9610) (Millipore) (1∶100), mouse anti-rat Nestin (clone rat401) (BD Bioscience) (1∶100), mouse anti-rat neuronal βIII-tubulin (clone Tuj1) (Babco) (1∶500), mouse anti-human CNPase (clone 11-5B) (Sigma Aldrich) (1∶200), rabbit monoclonal anti-Ki67 (clone Sp6) (Abcam) (1∶100), rabbit anti-human PDGFR-α (Cell Signaling #3164) (1∶100), rabbit anti-human PDGFR-β (Cell Signaling #3169) (1∶50), mouse anti-rat Map2 (HM-2) (1∶1000) (Sigma Aldrich), mouse anti-NeuN (clone A60) (Millipore) (1∶200), rabbit anti-Sox2 (Millipore) (1∶500), rabbit anti-Calbindin-D-28-K (EG-20) (Sigma Aldrich) (1∶2000) and rabbit anti-human cleaved Caspase-3 (Asp175) (Cell Signaling #9664) (1∶300). Secondary biotinylated antibodies included swine anti-rabbit IgG (Dako) (1∶500), donkey anti-rat IgG (1∶500), and donkey anti-mouse IgG (1∶500) (Jackson Immuno-Research).

## Results

### PDGF-A_L_ transgenic mice die at the median age of 120 days

To further explore PDGF-A_L_ function in the embryonic and adult brain, we generated transgenic mice in which expression of PDGF-A_L_ under control of the GFAP promoter was targeted to astrocytic cells of the developing and adult brain. Pronuclear injections of the hGFAPp-PDGFA_L_-IRESβGEO construct (Figure 1A) resulted in 5 transgenic founders. First, we used PCR analysis of DNA from tail biopsies to confirm the presence of the transgene (Figure 1B). Second, whole mount X-gal staining of brains verified activity of the transgene in one of five transgenic lines (line 8, A_L_8), with strong staining over the whole surface of the brain after birth (Figure 1C) and identical to results from hGFAPpLacZ reporter mice [23]. Third, sections from the brains were analyzed for transgene expression by IHC for β-gal demonstrating strong expression of the transgene in scattered cells all around the brain (Figure 1D-F). Particularly strong expression was seen in the ventricular wall (especially in the roof of the lateral ventricles), in corpus callosum, on the outer surface of the brain (glia limitans), and in the cerebellum (Figure 1E). No β-gal immunoreactivity was detected in wt brains. Another pronuclear injection was performed to create more PDGF-A_L_ overexpressing transgenic mice and confirm the observed phenotype. Founder #12 was found to possess the active transgene and β-gal staining of this mouse brain showed moderate transgene activity (data not shown).

**Figure 2:**
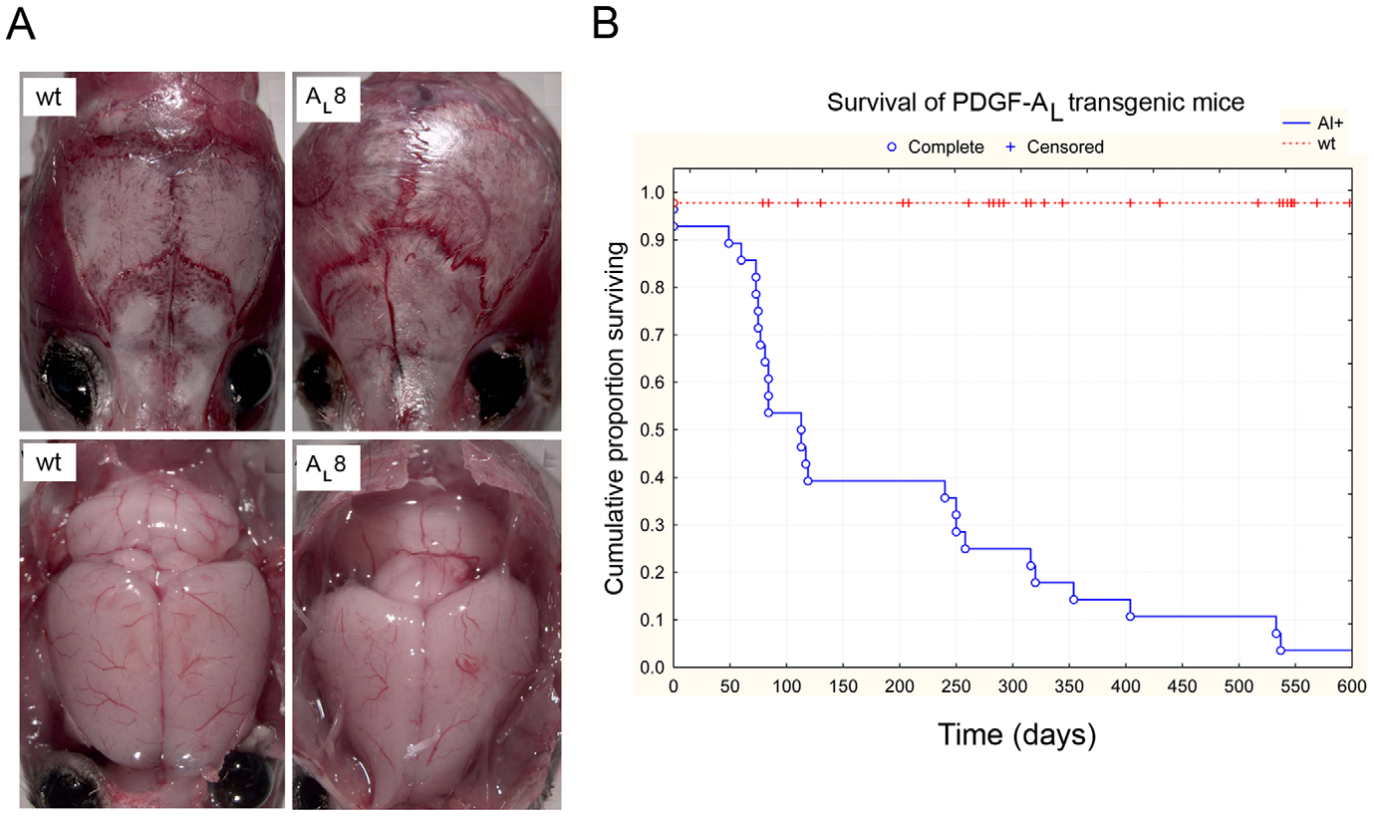
PDGF-A_L_ transgenic mice die from abnormalities in the brain. (A) Skull enlargement as exemplified in PDGF-A_L_ transgenic mice of line 8 (A_L_8). Compared to wt littermate controls there was an abnormal increase in the amount of fluid in the subarachnoid space situated between the brain surface and the skull in transgenic mice. (B) Kaplan-Meier survival curve showing the life span of transgenic mice that included data from 25 mice of line #8 and the founder mouse #12.

PDGF-A_L_ transgenic mice of line 8 were fertile and their offspring were vital at birth until the approximate age of one and a half month after which most of these mice developed significant skull enlargement and died (Figure 2A). Importantly, the founder #12 died at the age of 4 months with the same brain phenotype as line 8 mice. A systematic survival analysis was performed including 26 transgenic mice and non-transgenic wt mice, which were monitored on a daily basis. 25 of the 26 PDGF-A_L_ mice died or were sacrificed due to neurological symptoms and/or to skull enlargement with the overall median survival time of 120 days (Figure 2B). Founder of line 8, survived more than two years and was sacrificed due to old age. It was observed to have an atrophic cerebellum with thin granular layer (Table S1). Necropsy of mice from PDGF-A_L_ line 8 revealed an abnormal retention of fluid in the subarachnoid space between the brain surface and the skull. In addition, the pressure from this fluid resulted in deformation of the skull bones and made the surface of the brain become smooth, giving the brain an unusually compressed and thin shape (Figure 2A). All major organs of all the animals in this study were further examined and no abnormalities other than in the brain were found.

The reproductive ability of PDGF-A_L_ line 8 mice was compared to C57/Bl6 breeding couples. Line 8 (16 litters) showed no difference in mean litter size compared to wt breeding couples, 6.4 versus 7.6 pups per litter (p>0.05). The ratio between the number of transgenic progeny and the number of non-transgenic was 47.2% (p>0.05), hence no indication of embryonic lethality. We observed a slight difference in the sex ratio of progeny from the PDGF-A_L_ transgenic line 8 compared to the expected 1∶1 ratio in the hemizygous crossings. Sixteen litters were analyzed and there was a statistically significant but still relatively small increase in the number of male mice among the transgenic progeny (60% males versus 40% females, p<0.05, transgenic mice n = 50). Breeding of the founder mouse #12 with C57/Bl6 mice did not result in transgenic progeny.

### Locally increased cellularity in the brains of PDGF-A_L_ transgenic mice

**Figure 3:**
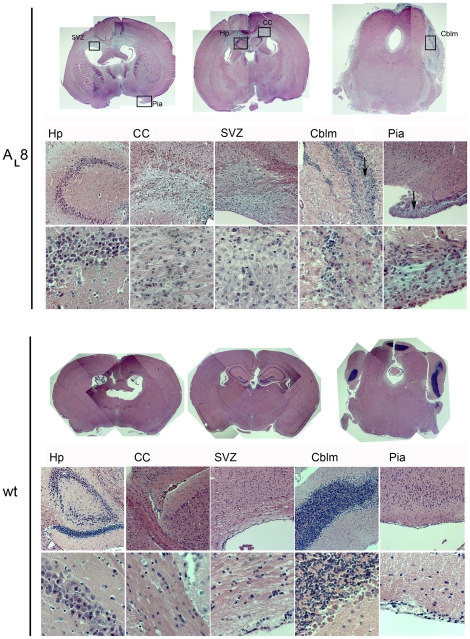
Increased cellularity in PDGF-A_L_ brains. An overview of coronal sections of a PDGF-A_L_ transgenic mouse brain (A_L_8 line, #4012) and wt littermate control (H&E staining). Higher magnification images in lower panels of selected areas from the overviews serve to illustrate areas of increased cellularity (20x and 40x objectives, respectively). A rather loose texture of the accumulating “clear” cells is indicated with arrows in pia and cerebellum. Strikingly, the borders of the inner granular layer in cerebellum appeared to be diffuse compared to wt control brain. Abbreviations: Hp-hippocampus, CC-Corpus callosum, SVZ- subventricular zone, Cblm – cerebellum.

**Figure 4:**
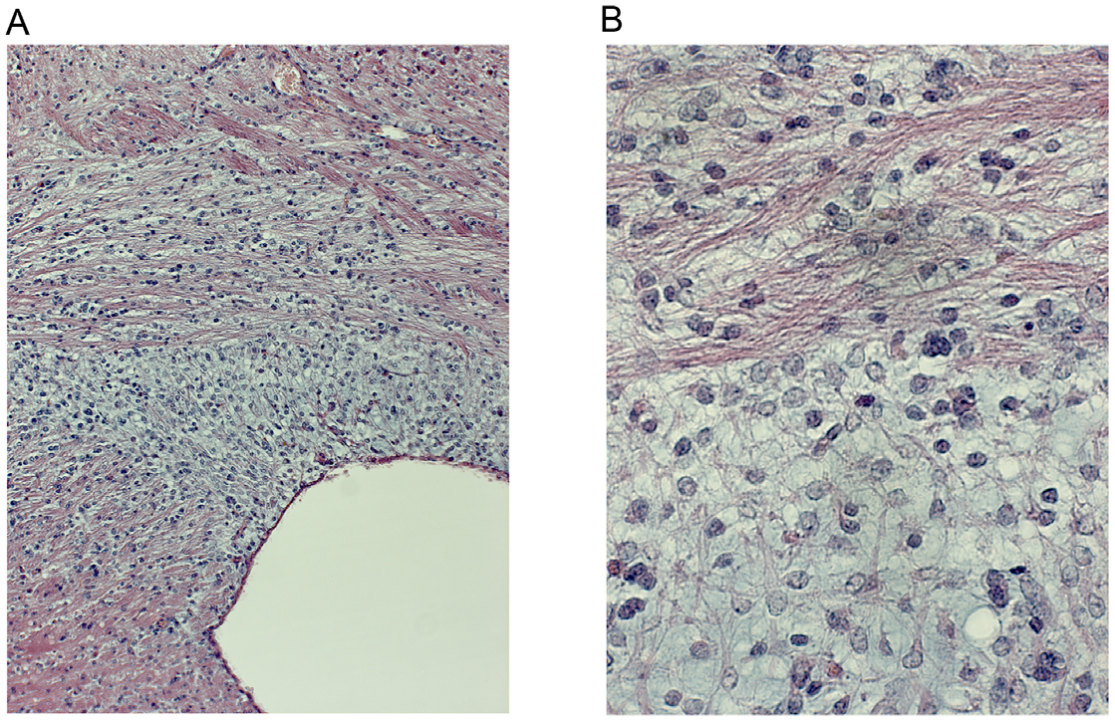
Morphology of accumulating cells in the PDGF-A_L_ brain. Light microscopy images of an H&E stained section, in low (A) and high (B) magnification, illustrating the presence of immature cells in the roof of the lateral ventricle in a PDGF-A_L_ transgenice mouse brain (A_L_8 line, #4012).

Brains from PDGF-A_L_ and wt mice were further analyzed by routine histology. Sagittal and coronal sections stained with Hematoxylin-Eosin revealed areas with highly increased cellularity in the wall and especially the roof of lateral ventricles and their position was mostly subependymal (Figure 3). These cells were characterized by their clear cytoplasm and small, rounded nuclei and hence based on this morphology we denoted them as “clear cells”. These cells are shown in greater detail in Figure 4. In some sections, a fraction of the cells were seen breaking into the ventricle through the ependyme, leading to seeding of cells on the ventricular lining, even as far as the 4^th^ ventricle. A majority of the mice that died or were sacrificed due to symptoms displayed locally increased cellularity in the brain although in varying amounts and distribution (Table S1). In most of the brains, the abnormal cells were also found within the corpus callosum and along its extensions, in the thickened pial lining forming pial outgrowths and in the medulla of cerebellum (Figure 3). In cerebellum, the inner granular layer appeared diffuse and disorganized when compared to wt brains. This was due to the presence of “clear” cells expanding from the white matter into the granular layer (Figure 3). “Clear” cells were also present in the white matter of pons and the brain stem in some mice. Essentially, the distribution of these abnormal “clear” cells overlapped with the distribution of high transgene expression visualized by β-gal IHC.

**Figure 5:**
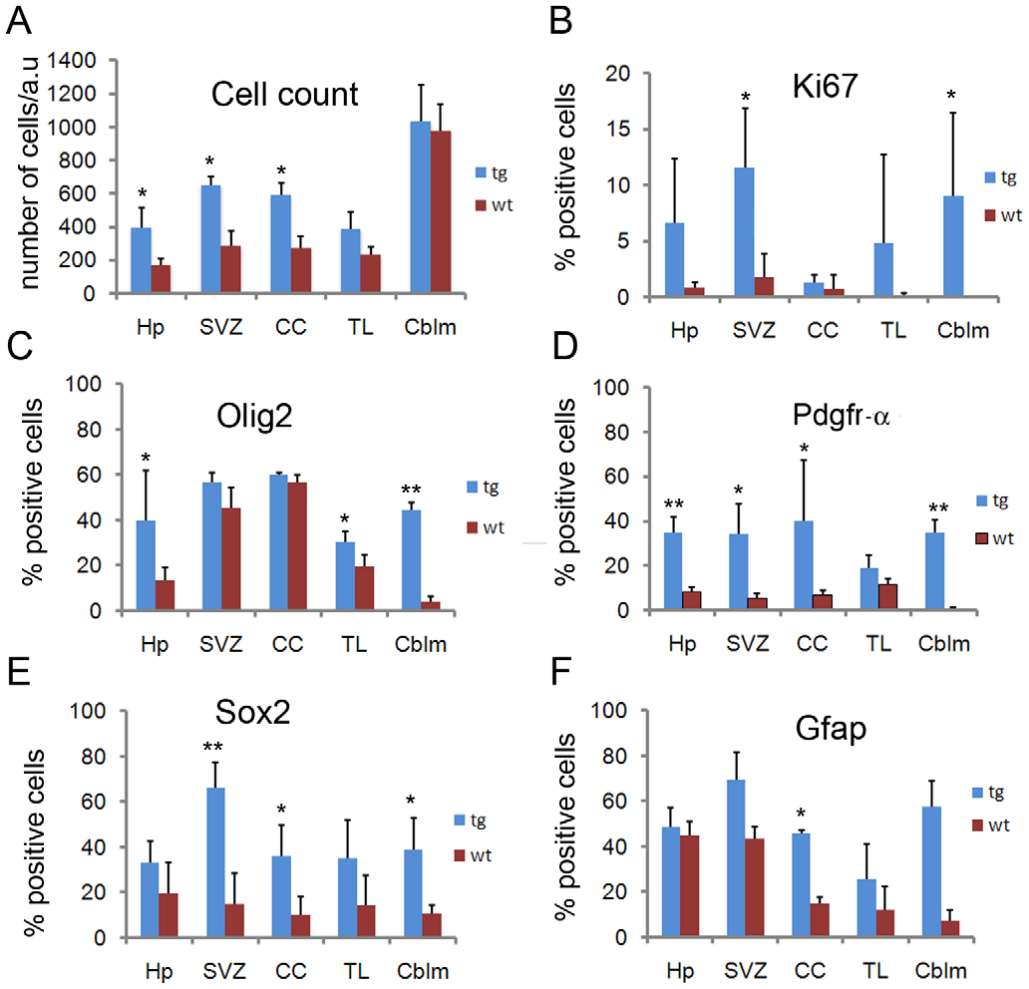
Quantification of cell numbers and of cells expressing different lineage markers in brain areas with increased cellularity. (A) Quantification of total cell numbers in hippocampus (Hp), subventricular zone (SVZ), corpus callosum (CC), temporal lobe area (TL), and cerebellum (Cblm). The counting is based on the comparison of one high power field (40x obj.) from each anatomical region in transgenic (A_L_8) and wt mice. Mean and standard error of the mean is presented for 5 transgenic mice (A_L_8) and 5 wt mice. Differences at p<0.05 were considered significant and are marked with (*). Given that the protruding lesions in Pia did not have a corresponding wt area to count it was excluded from the analysis (a.u =  area unit). (B–F) Frequencies of Ki67, Olig2, Pdgfr-α, Sox2, and Gfap immunoreactive cells in wt and transgenic mice. Quantification was based on the counting of both the total number of cells and positively stained cells for each individual marker in one high power field (40x obj.). Mean and standard error of the mean is presented for 3 transgenic mice (A_L_8) and 3 wt mice. Differences at p<0.05 were considered significant and are marked with (*). P-values<0.01 are marked with (**).

To substantiate the histological findings we counted the numbers of all cells in one selected high magnification area corresponding to each of the above mentioned regions from five transgenic mice brains and compared this to the cell counts from similar areas in five wt brains. A statistically significant increase in numbers of cells was seen in the lateral ventricle wall, hippocampus and corpus callosum (p<0.05 for each of these regions), whereas no significant difference in cell number was found in the cerebellum or temporal lobe area (p>0.05) (Figure 5A). The subgranular zone of the dentate gyrus in hippocampus was morphologically intact, although the hippocampal area as a whole, including white matter, showed an increase of cells (data not shown).

In summary, our results demonstrate that PDGF-A_L_ can induce abnormal accumulations of cells in the mouse brain, similar to what was seen when PDGF-A_S_ was injected directly into the lateral ventricle of mice [31]. In addition, we observed accumulation of translucent fluid in the subarachnoid space and skull enlargement indicating an increased intracranial pressure contributing to the lethality of the lesions. Choroid plexus from transgenic mice showed no changes in structure compared to wild type mice. A slight to moderate increase in the ventricle size was noted in a subset of the PDGF-A_L_ transgenic mice but the cerebrospinal-like fluid accumulated mostly in the subarachnoid space and seemed to compress the brain from the outside.

### Increased proliferative activity in the brains of PDGF-A_L_ transgenic mice

We next assessed the level of cell proliferation in transgenic mouse brains by using the Ki67 antibody. Clearly, regions with accumulating cells displayed an increased percentage of cells that stained positive for Ki67 (Figure 5B). For instance, in the subventricular zone (SVZ), the fraction of Ki67+ cells turned out to be 11.6±5.5 (%) in transgenic mice versus 1.73±2.1 (%) in wt (n = 3/genotype; p<0.05). We could also detect more Ki67 positive cells in the hippocampus area, in the pial outgrowths and in cerebellum of PDGF-A_L_ mice (Figure 5B) (Table S2). In wt mice, a small localized area of intensively proliferating cells was observed in the lateral ventricular wall in a region that may correspond to the neural stem cell niche, whereas almost no proliferation was observed in the roof of the lateral ventricles, in pia and cerebellum. In transgenic mice, the proliferating cells were widely scattered in the roof of the lateral ventricles which was different from the organized pattern of Ki67-positive cells observed in the lateral ventricular wall. We conclude that the regions with accumulating cells in the PDGF-A_L_ transgenic mouse brain displayed significantly high proliferative activity.

### Increased fractions of Pdgfr-α and Olig2 positive cells in the brains of PDGF-A_L_ transgenic mice

**Figure 6:**
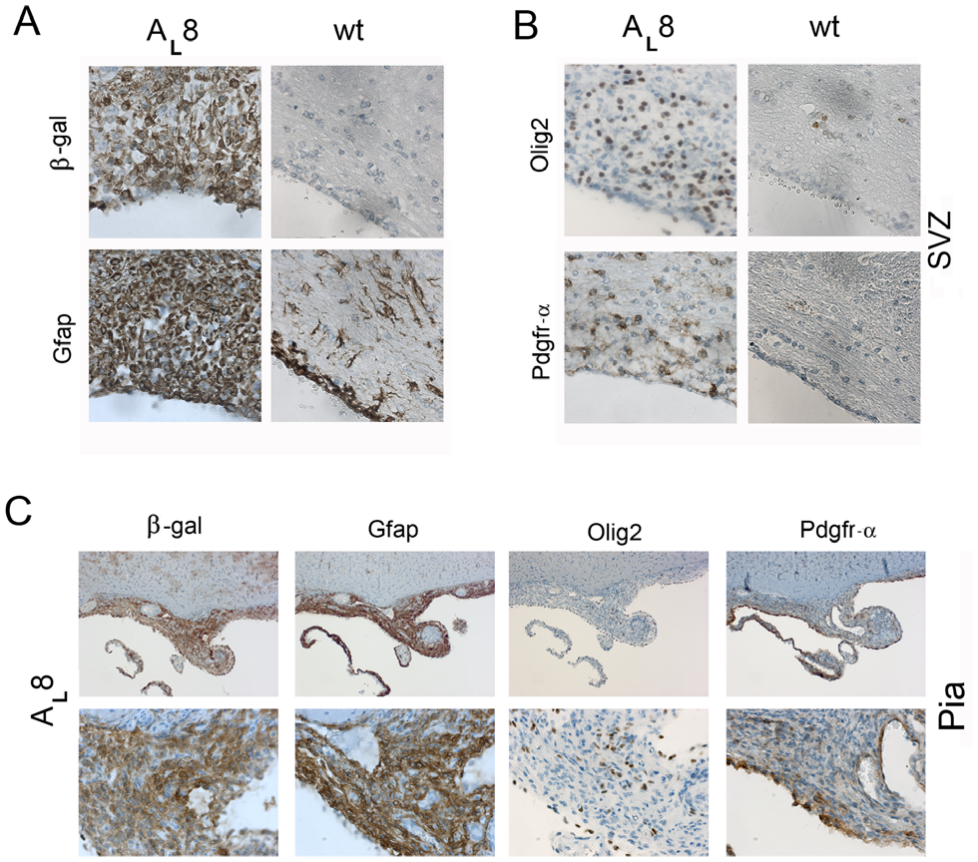
Presence of different lineage marker proteins in brain areas with increased cellularity. (A) IHC for β-gal and Gfap in the SVZ from an A_L_8 mouse (#4012). The stainings revealed strong expression of the transgene (β-gal marker) and the location of Gfap^+^ astrocyte-like cells matched the expression of β-gal in parallel sections. Corresponding SVZ areas are shown for wt control brain (40x obj.). (B) Presence of Olig2 positive and Pdgfr-α positive cells in the SVZ (40x obj.) (C) IHC for β-gal, Gfap, Olig2 and Pdgfr-α in a pial lesion of a transgenic mouse of line A_L_8 (#4016). Abbreviation: SVZ-subventricular zone.

Immunohistochemistry was carried out using a variety of cell type-specific markers to further characterize the nature of the accumulated cells. We first observed that the β-gal expression was particularly strong in areas with increased cellularity strongly indicating that this was caused by PDGF-A_L_. Areas with high expression of β-gal displayed strong Gfap expression (Figure 6A and 6C), and both Gfap+ cells and β-gal+ cells displayed an astrocyte-like morphology (Figure 1F). Next, we compared the patterns of Gfap and Olig2 expression in the SVZ and pia (Figure 6A-C). Olig2, which is present in oligodendrocyte progenitors, and serves as a marker for human gliomas, was increasingly expressed in areas with accumulation of cells in the PDGF-A_L_ brains (Figure 6B and 6C). Also the pial proliferations consisted of Gfap+ as well as Olig2+ cells indicating their neural character (Figure 6C). A significant increase in the number of Olig2+ cells was seen in cerebellum and hippocampus of transgenic mice (p<0.05) (Figure 5C) but the difference in Olig2+ cells between wt and transgenic mice did not reach statistical significance in SVZ and corpus callosum. However, when all the selected areas were taken together, 46.2±5.1 (%) of cells were Olig2+ in transgenic mice compared to 27.7±1.4 (%) Olig2+ cells in wt mice (n = 3/genotype; p<0.05). The mature oligodendrocyte protein CNPase was not expressed in the accumulating cells and stainings for neuronal lineage differentiation markers such βIII-tubulin and Map2 were mostly negative. We noticed that the subgranular zone cells of the dentate gyrus in hippocampus strongly expressed both the transgene and Gfap, but in the absence of any histological abnormality in this area (data not shown).

**Figure 7:**
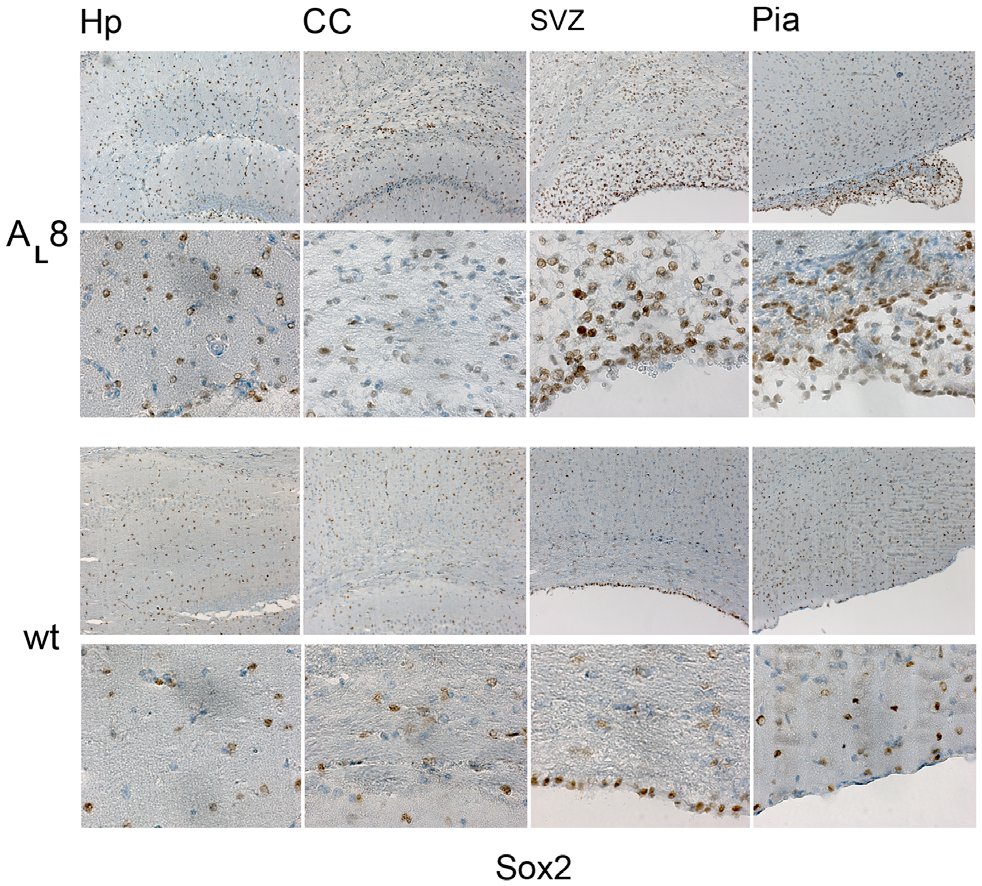
Sox2 positive cells in the areas with increased cellularity. Presence of Sox2+ cells in different regions of a transgenic mouse brain (A_L_8, # 4012), as shown in the upper two panels in low (20x obj.) and high magnification (40x obj.), respectively. Shown are also corresponding regions in a wt brain in the lower two panels (20x and 40x obj.). Abbreviations: Hp-hippocampus, CC-Corpus callosum, SVZ-subventricular zone.

We analyzed the sections further by staining for Pdgfr-α. Of note, Pdgfr-α is normally expressed by cells in the meninges and by many tissues outside the CNS [32]. This explains some of the positive staining seen in wt tissue in the meninges. In the wt mouse brain, Pdgfr-α was also present on scattered cells in the brain stem presumably representing normal oligodendrocyte progenitors. In the transgenic mice, we found an increased Pdgfr-α staining, and positive cells were frequently scattered around the brain with strong signals in the regions with increased cell numbers (Figure 6B and 6C). For instance, the frequency of Pdgfr-α+ cells in the SVZ of transgenic mice was 34.3±13.5 (%) versus 5.4±2.2 (%) in wt mice (n = 3/genotype; p<0.05) (Figure 5D). Next we carried out IHC for detection of the putative neural progenitor cell marker protein Sox2 (Figure 7). Scattered Sox2+ cells could be seen in regions of increased cellularity and were significantly more frequent in the SVZ of transgenic mice, namely 66.9±11.2 (%) in transgenic mice compared to 14.7±13.6 (%) in wt mice (n = 3/genotype; p<0.05). Indeed, in a wt mouse the thin line of Sox2+ cells was more strictly ependymal (Figure 7). Sox2+ cells were also detected in the pial outgrowths of the transgenic mice (Figure 7).

### Abnormal morphology of the cerebellum

**Figure 8:**
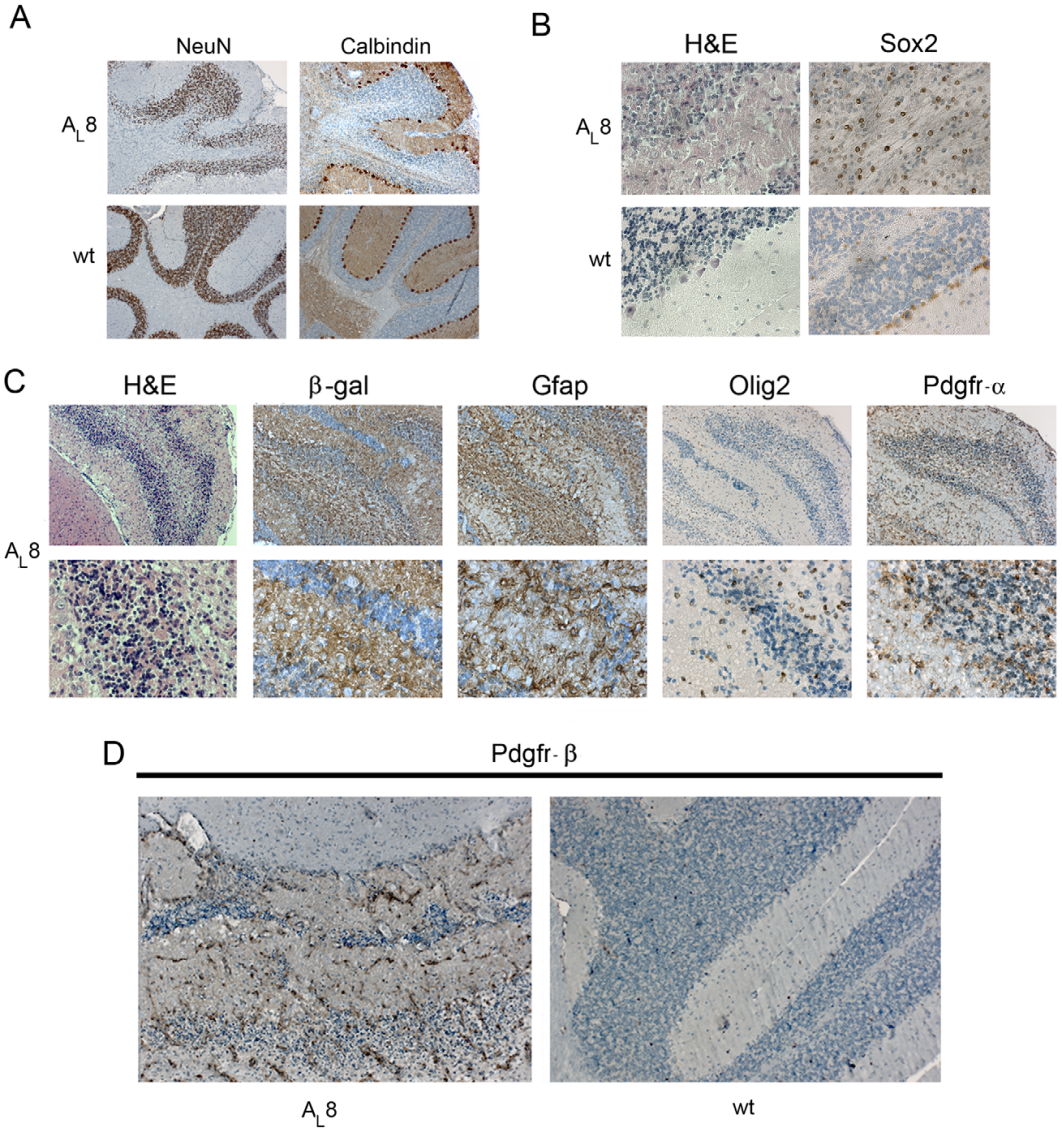
Changes in the presence of different cell lineage marker proteins and in the structure of cerebellum in PDGF-A_L_ transgenic mice. (A) IHC for Calbindin (marker for Purkinje cells) and NeuN (marker for terminally differentiated neuronal cells, here granular cells) in the cerebellum of the transgenic mouse A_L_8, #4016 (upper panel) and in cerebellum of a wt mouse (lower panel). (B) H&E staining and expression of Sox2 in cerebellum of a transgenic brain (A_L_8, #4012) compared to corresponding areas in a wt cerebellum (40x obj) (C) H&E staining and IHC for β-gal, Gfap, Olig2 and Pdgfr-α in cerebellum from a transgenic mouse (D) IHC for Pdgfr-β cerebellum of a transgenic (A_L_8 #4011) and a wt mouse (20x obj.).

Special attention was given to the morphology of the cerebellum in order to determine the amplitude of disruption caused by PDGF-A_L_. As mentioned, the structure of the cerebellum was deformed when compared to wt mice (Figure 3). Using the markers NeuN for mature granule cells and Calbindin for Purkinje cells we could determine that the layers of the cerebellar cortex maintained their correct order in relation to each other, but due to the expansion of abnormal cells into the inner granular layer this became disintegrated (Figure 8A). In addition, IHC for β-gal, Gfap and Olig2 showed strong positivity in the cerebellum of transgenic mice (Figure 8C) and this was confirmed by cell counting (Figure 5 and Table S2). For instance, the frequency of Olig2+ cells per unit area in cerebellum of transgenic mice was a remarkable 44.5±3.4% when compared to only 3.8±2.6 (%) in wt mice (n = 3/genotype; p<0.05) (Figure 5C). In addition, the fractions of Pdgfr-α+ and Sox2+ cells were increased (Figure 5D and 5E). Of note is the distinct 1-2 cell layer thick band of well-organized Sox2+ cells at the level of Purkinje cell bodies observed in wt brains but not in transgenic mice, further illustrating the disintegrated structure of cerebellum (Figure 8B). Finally, Pdgfr-β IHC revealed an increased amount of capillary vessels especially in the cerebellum of the transgenic mice compared to wt littermates (Figure 8D). The vascular structures were thick-walled compared to that seen in wt mice. In comparable sections of the wt brains, Pdgfr-β was present only on a few discrete cells, viz pericytes tightly associated with capillary vessels.

### The abnormal cell populations in areas of transgene expression constitute in part Pdgfr-α positive precursors with similarities to oligodendrocyte progenitors

**Figure 9:**
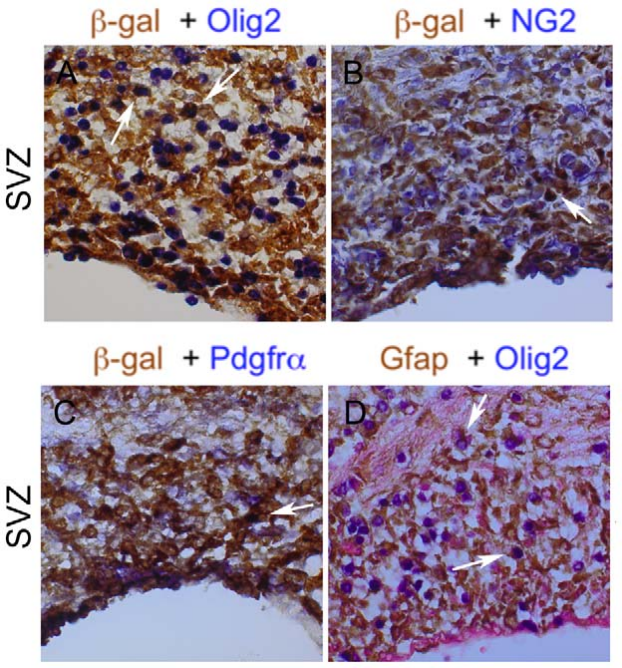
Accumulated cells expressing oligodendrocyte progenitor markers do not generally overlap with but are mixed with β-gal and Gfap positive cells. (A–C) Co-immunostainings for β-gal (brown color) with Olig2, Pdgfr-α and NG2 (blue color) respectively. Only a fraction of the accumulated cells showed double positivity as indicated with arrows in β-gal/Olig2 double IHC (see text for details). (D) Co-immunostaining (IHC) of Gfap (brown) and Olig2 (blue). In D the section was also counterstained with Fast Red dye (pink) (40x obj.). The tissue sections represent the SVZ from A_L_8 mice (#4007 and #4008).

Oligodendrocyte progenitors usually co-express Pdgfr-α, the transcription factor Olig2 and the neuroglial chondroitin sulfate proteoglycan NG2. To further investigate the nature of the additional cells in transgenic mice we carried out a series of co-stainings by IHC for β-gal in combination with Olig2, Pdgfr-α or NG2, as well as co-stainings for Gfap and Olig2. Interestingly, as is here exemplified with the SVZ in the roof of the lateral ventricle, there was an evident non-overlap between β-gal expressing cells and those cells that stained positive for Olig2, NG2 or Pdgfr-α, respectively (Figure 9A–C). We estimated the fraction of β-gal+ cells that were co-expressing Olig2 by analyzing and counting cells in high power magnification fields. The predominant nuclear Olig2 staining made it most suitable for this analysis. In the SVZ on average 50±20 (%) of β-gal+ cells expressed Olig2 (tg mice: n = 3). Moreover, only ∼30±10 (%) of the β-gal+ cells in hippocampus expressed Olig2 (tg mice: n = 3). Similarly, the numbers of cells co-expressing β-gal and Pdgfr-α in the SVZ and hippocampus was only on ∼10% in each location. In support of the notion that cells expressing the transgene might be a different population of cells than those expressing Olig2 and/or Pdgfr-α we found that the fraction of Gfap+ cells that co-expressed Olig2 was only ∼10±4 (%) in the SVZ, and ∼10±7 (%) in the hippocampus (tg mice: n = 3) (Figure 9D). In conclusion, the IHC analyses revealed that a substantial fraction of the accumulated cells most likely represent oligodendrocyte progenitors that stain positive for Pdgfr-α, Olig2 and NG2. These cells were mostly different from, but intimately mixed with, the astrocytic, transgene expressing cells.

### The most extensive brain lesions display features of neoplasia

**Figure 10:**
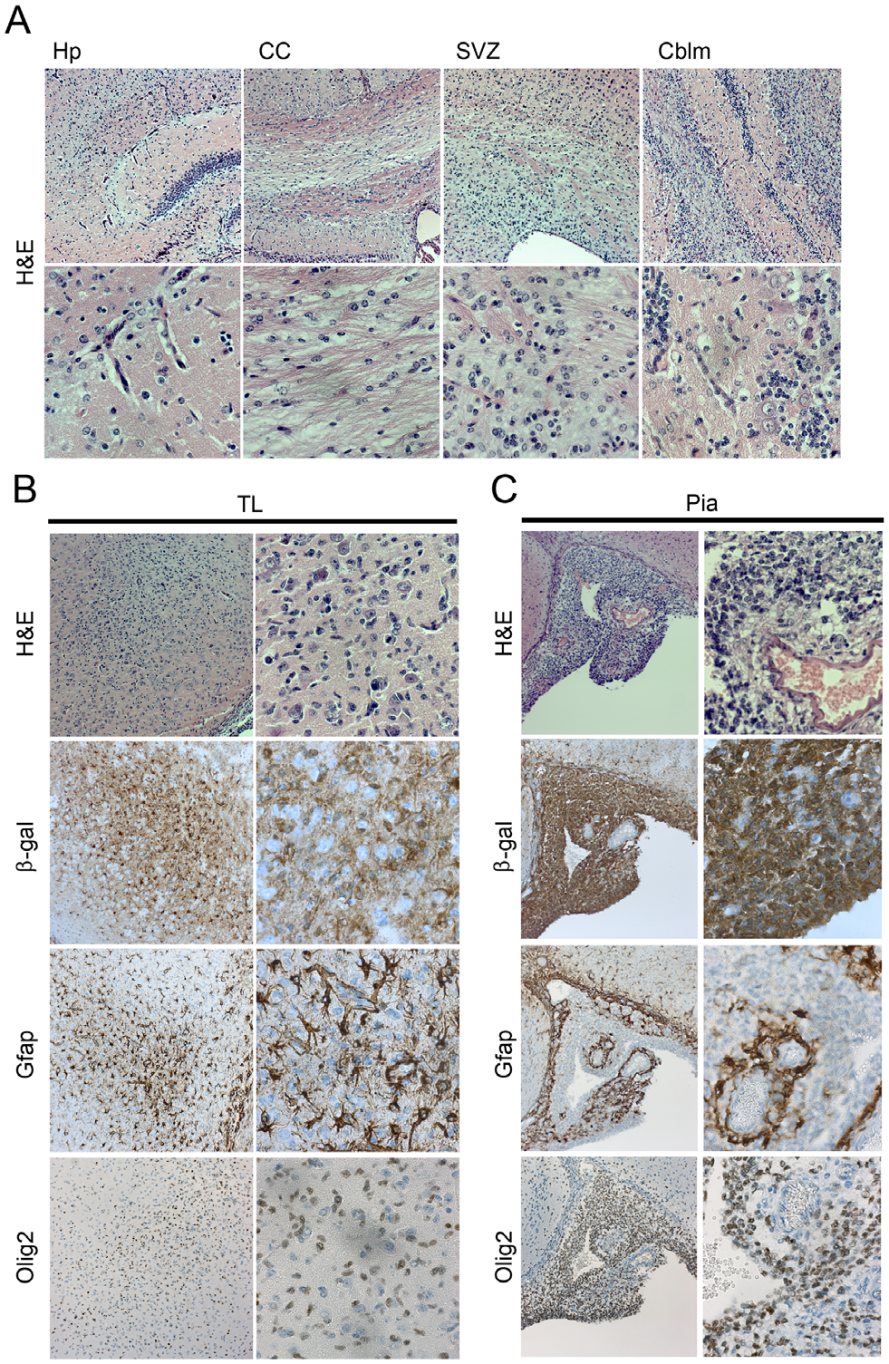
Lineage marker protein expression in brain areas with glioma-like lesions. (A) Different areas of a transgenic mouse brain with neoplastic glioma-like lesions (A_L_8, #4000) as indicated (20x obj. upper panel and 40x obj. lower panel, H&E staining). (B) IHC for β-gal, Gfap and Olig2 in the temporal lobe area of the same transgenic mouse brain as in (A). (C) IHC for β-gal, Gfap and Olig2 in the pial area of transgenic mouse brain A_L_8, #4000 (20x obj. left panels and 40x obj. right panels). Abbreviations: Hp-hippocampus, CC-Corpus callosum, SVZ-subventricular zone, TL-temporal lobe.

**Figure 11:**
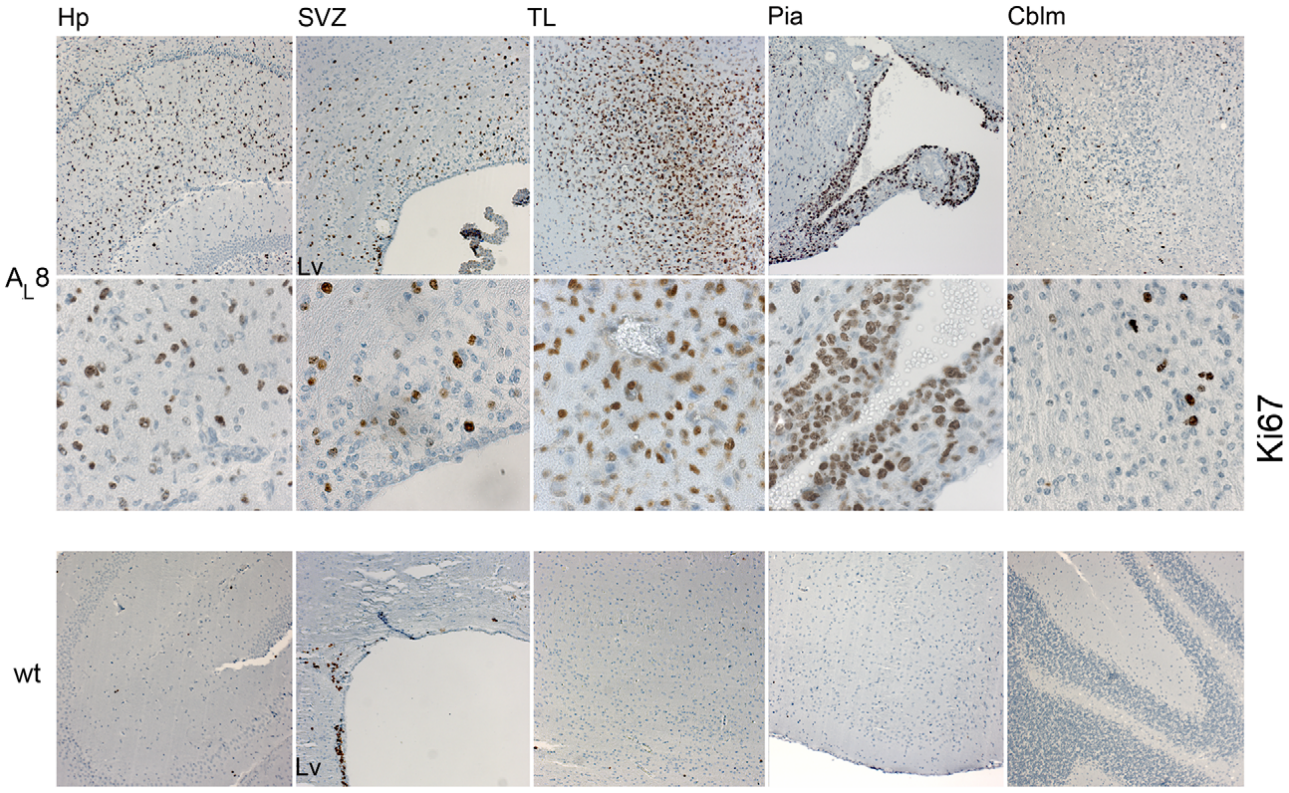
Ki67 in different areas of a transgenic mouse brain with glioma-like lesions. IHC for Ki67 in different areas of a transgenic mouse brain (A_L_8, #4000) compared to similar areas in a wt mouse brain. Abbreviations: Hp-hippocampus, SVZ-subventricular zone, TL-temporal lobe, LV-left ventricular wall, and Cblm-cerebellum.

Four of the transgenic brains showed more extensive lesions. As exemplified with the worst case (mouse #4000) there was a heavy diffuse infiltration of cells in virtually the whole brain, encompassing most white matter areas including the hippocampal and cerebellar outer layers and this was associated with abundant, angulated and thick-walled capillary structures (Figure 10A). The pial proliferations were extensive, forming cell rich lumps with obvious mitotic (Figure 11) and abnormal cells (Figure 10C), in a few areas with a bluish tint and microcystic fluid accumulations. Where these lumps were present on the pial surface the glia limitans was disrupted (Figure 10C). Also the temporal lobe was especially cell-rich, with a localized area of cells carrying uneven, multi-lobulated nuclei and at the same time a local increase in Gfap, Olig2 and Pdgfr-α positive cells (Figure 10B) (Table S2). Stainings for Map2 and βIII-tubulin revealed only sparse, dislocated neuronal cells in the same area (data not shown). Moreover, staining for Ki67 revealed massive proliferation in this mouse brain in several different anatomical regions (Figure 11) (Table S2). Locally, in the neoplasia-like area of the temporal lobe, the Ki67 labeling index was as high as 53%, whereas it was only 1.4% in the corresponding area from a wt brain and 1.2% in another PDGF-A_L_ brain (Table S2). In summary, the analysis of cells in the neoplasia-like areas revealed accumulation of highly proliferative abnormal glial precursor cells displaying Gfap-positivity and frequent Olig2 and Pdgfr-α positivity coupled to a high proliferation rate. Given the appearance of mixed glial cell phenotypes, diffuse spread and increased vascularity these areas are similar to human oligoastrocytomas grade III (WHO scale).

## Discussion

In this study we overexpressed PDGF-A_L_ under the astrocyte specific promoter for *GFAP*. As mentioned this promoter is active in mature astrocytic cells as well as in neural stem cells of the lateral ventricle wall. In the CNS, PDGF-A is normally expressed by many neurons as well as by astrocytes [21], [33], [34], but these cells are not generated in large numbers until after birth. In our study we created additional sources of constant PDGF-A expression with fatal consequences. The GFAPp-PDGF-A_L_ mice showed a distinct phenotype with accumulation of cells in many different locations in the brain where the transgene was active, as well as accumulation of a thick translucent fluid in the subarachnoid space. The choroid plexus of the lateral ventricle produces most of the cerebrospinal fluid [35]. Fluid is normally produced continuously at a rate sufficient to fill the ventricular system together with cranial and spinal subarachnoid spaces several times a day [35], [36]. Most cases of hydrocephalus in humans are caused by blockage of the flow of cerebrospinal fluid. The fluid fills the ventricles and compresses the brain from within leading to a rise of the intracranial pressure and expansion of the ventricles and [37]. Choroid plexus from PDGF-A_L_ transgenic mice showed no changes in structure compared to wt mice, but a slight to moderate increase in the ventricle size was noticed in a subset of the PDGF-A_L_ transgenic mice. Enlarged lateral ventricles have recently been described in transgenic mice expressing PDGF-B under control of a minimal nestin enhancer element [38]. However, in the present mice fluid accumulated mostly in the subarachnoid space and to the extent that it compressed the brain from the outside. The observed accumulation of fluid together with skull enlargement and compression of the brain indicated that an increased intracranial pressure contributed to early death of the animals.

In the PDGF-A_L_ transgenic mice we detected strong expression of Gfap in the brain, which mostly followed the same pattern as that of β-gal (the transgene reporter), indicating that PDGF-A_L_ was produced by astrocytic cells. As PDGF-A_L_ has a C-terminal tail that helps to associate the protein with the extracellular matrix and the surface of the producing cells [39], the PDGF-A_L_ site of action may be mostly limited to the secretory cell itself and its closest neighbors. Indeed, we detected increased numbers of Pdgfr-α carrying cells in the areas with β-gal+ cells indicating that the introduced PDGF-A_L_ had stimulated responsive cells. It has previously been shown that Pdgfr-α is expressed by proliferative and migratory glial progenitor cells, called O-2A progenitors [40], [41]. These cells originate in the ventricular and subventricular zones of the embryonic brain and spinal cord, then proliferate and migrate throughout the developing CNS [42]. They start to develop into myelinating oligodendrocytes around birth, continue to divide and differentiate during early postnatal life. At the same time Pdgfr-α is rapidly downregulated after progenitor cells stop dividing and start to differentiate [43]. Thus, normally the distribution of Pdgfr-α positive cells in the embryonic and postnatal brain reflects the distribution of O-2A progenitors, rather than that of differentiated oligodendrocytes. During development, O-2A numbers are limited by the supply of PDGF-A. It was demonstrated that in mice overexpressing PDGF-A in neurons (neuron specific enolase (NSE)-PDGF-A_S_ mice), the number of oligodendrocyte progenitor cells increased 7-fold. It led to secondary overproduction of oligodendrocytes, many of which were situated in for them abnormal locations. However, at an early stage of development, the extra cells were all eliminated by programmed cell death, leaving the final number and distribution of mature oligodendrocytes within the normal range [16]. No special phenotype of adult mice was reported in that study. However, a phenotype in adult mice has been reported in another study, where under control of the GFAP promoter, PDGF-A_S_ was found to regulate oligodendrocyte progenitor number in adult CNS, demonstrating an increase in their number [44], similar to what is observed here. In fact, in the transgenic mice presented here we detected virtually no apoptotic figures among the accumulated cells by staining for cleaved caspase-3 (not shown), and cell numbers were in general not adjusted with time. Our transgenic mice phenotype indicates that also PDGF-A_L_ is a potent factor in postnatal brain development. The results are in accordance with other studies in which the *Pdgfa* gene was disrupted [22], or in studies where PDGF-A_S_ was overexpressed, leading to a decreased or increased number of oligodendrocyte progenitors, respectively [16]. However, it should be kept in mind that expression of PDGF-A_S_ mRNA is predominant over PDGF-A_L_ in the normal brain [16].

What is then the origin and significance of the extra cells found in the brains of PDGF-A_L_-transgenic mice and of the neoplastic lesions? We could consider an origin from adult neural stem or progenitor cells which normally display astrocytic features, as well as from oligodendrocyte progenitor cells or even astrocytes. The strong Pdgfr-α expression in PDGF-A_L_ brains indicates that a fraction of the accumulating cells represent some type of progenitors rather than mature oligodendrocytes, which is in line with the absence of CNPase in these cells. In order to obtain support for this possibility we stained parallel sections with Olig2 and NG2 antibodies demonstrating increased expression of these proteins. A majority of the cells that stained positive for Olig2 or Pdgfr-α did not express Gfap or the transgene marker. Thus, a fraction of the accumulated cells can therefore be defined as oligondendrocyte progenitors/O-2A progenitors. This finding is in agreement with the known ability of PDGF-A to stimulate expansion of oligodendrocyte progenitors. Other studies have shown that PDGF-A can bias cell-fate decisions, for example mouse neural progenitors residing in the SVZ are induced to become OPCs rather than neurons in response to PDGF treatment [31]. Indeed, PDGF-A treatment delays differentiation of neural precursors that remain in an immature state [45]. It has also been known for quite some time that PDGF-A has a dedifferentiating effect on Gfap expressing astrocytes [46]. Thus, PDGF-A has profound effects on neural cell proliferation and differentiation that may be of importance during glioma development [47].

However, we did also detect more frequent Gfap+ cells mixed with the oligodendrocyte progenitors. Gfap expression is a common characteristic of normal subependymal cells [48]. This layer contains adult neuroepithelial stem cells, oligodendrocyte and astrocyte progenitor cells [48]. The strong expression of Gfap and Olig2, mostly in different cells but also infrequently in the same cells, as well as the SVZ/subependymal location of the abnormally accumulating cells in PDGF-A_L_ transgenic mice speaks in favor of their potential derivation from neuroepithelial stem cells. One might also speculate that PDGF-A_L_ stimulates expansion of a Gfap+ glial progenitor/stem cell population that in turn gives rise to progeny representing more differentiated astrocytic cells as well as oligodendrocyte progenitors.

Interestingly, our observations support an overt neoplastic nature of some of the lesions, as seen in ∼20% (4/22) of the mice. Cells accumulated to form lumps both within the cerebral tissue and in pia, in these worst cases with high cell density, mitoses and nuclear atypia as well as locally increased amounts of cells expressing Pdgfr-α, Olig2 and Gfap. The increased proliferation and turnover of cells was indicated by locally high Ki67 labeling index accompanied by detectable clusters of cleaved caspase-3 positive cells. In support of our finding, it was reported that Pdgfr-α type B cells in the SVZ can be induced to form reversible hyperplastic glioma-like growths in response to PDGF-A infusion in the lateral ventricle [31].

While it is undisputable that PDGF induces glioma formation in mice it has remained debated to what extent the tumors represent pure oligodendrogliomas [47]. Indeed, retroviral expression of PDGF-B in newborn mice leads to oligodendroglioma-like tumors [49], whereas expression of PDGF-A in Gfap+ cells also induces rare mixed oligoastrocytomas in mice [46]. As another example, the inactivation of the Retinoblastoma tumor suppressor (Rb) in Gfap expressing cells led to perinatal lethality and astrocytoma development in mice [50]. In the transgenic mice presented here, we have diagnosed the lesions as resembling human anaplastic oligoastrocytomas WHO grade III because the neoplastic areas had a marked Gfap+ astrocytic component, showed a diffuse growth pattern and was accompanied by vascular proliferations, similar to WHO grade III human gliomas. The neoplastic lesions were not similar to grade IV glioblastomas and did not display pallisading cells, necrosis or glomeruloid vascular structures.

In summary, this work indicates that overexpression of PDGF-A_L_ in brain has effects which differ in some important aspects from previously reported studies on overexpression of PDGF-A_s_ and of PDGF-B on a wild type background and in the worst cases results in glioma-like lesions. However, it should be kept in mind that because of differences in the promoters used and in transgene copy numbers any direct comparisons between these transgenic lines remain difficult. Further studies will be required to elucidate the specific mechanisms by which overexpression of PDGF-A_L_ in brain promote a severe lethal phenotype, including neoplastic lesions.

## Supporting Information

Table S1 Summary of brain phenotypes in GFAPp-PDGF-AL transgenic mice.
(DOC)

Table S2 Frequency (%) of Pdgfr-α, Ki-67, Olig2, Sox2 and Gfap positive cells in individual brains of PDGF-AL transgenic and wt mice.
(DOC)
