# Supplementary material for: Brain Abnormalities and Glioma-Like Lesions in Mice Overexpressing the Long Isoform of PDGF-A in Astrocytic Cells
Source: PLoS One. 2011 Apr 7;6(4):e18303. doi: 10.1371/journal.pone.0018303 (PMC3072383; doi:10.1371/journal.pone.0018303)
Supplement: Table S1 — Summary of brain phenotypes in GFAPp-PDGF-AL transgenic mice. (DOC) [file pone.0018303.s001.doc]

| **MouseID** | **Age (months)** | **Enlarged skulls** | **SVZ** | **CC** | **Cbrm** | **Pia** | **Pons & BS** | **Cblm** | **Other comments** |
| --- | --- | --- | --- | --- | --- | --- | --- | --- | --- |
| 4000 | ~1.5 | + | ++ | +++ | + | +++ | NA | +++ | Large tumor-like lesions in pia, cell rich with visible mitoses. Diffuse proliferation of atypical cells. Increased capillary vessels in white substance, especially in temporal lobe areas of the brain. Glioma-like areas. |
| 4024 | ~2 | + | + | - | + | - | - | + | Found dead (frozen brain). |
| 4001 | ~2 | + | - | - | ++ | + | NA | NA | Found dead (frozen brain). |
| 4002 | ~2 | + | - | - | - | + | - | + | Found dead (frozen brain). |
| 4003 | ~2 | + | ++ | +++ | +++ | + | - | NA | Diffuse distribution of abnormal cells, around the whole ventricle, further laterally in the temporal/occipital lobe and including hippocampus. Diffusely infiltrating tumor-like lesion with some nuclear atypia and prominent capillary vessels. Glioma-like areas. |
| 4004 | ~2 | + | - | ++ | - | - | + | ++ | Found dead (frozen brain). |
| 4005 | ~2 | + | NA | NA | NA | NA | NA | NA | Found dead (brain not suitable for IHC). |
| 4006 | ~2 | + | NA | NA | NA | NA | NA | NA | Found dead (brain not suitable for IHC). |
| 4007 | ~2 | + | ++ | ++ | - | +++ | NA | ++ | Large clear cells in the pia with fluid- like content. Slight increase in capillary vessels in hippocampus area. |
| 4008 | ~2 | + | +++ | ++ | - | ++ | NA | ++ | Wide ventricles. Increased cellularity and prominent capillary vessels in hippocampus. |
| **ID** | **Age (months)** | **Enlarged skulls** | **SVZ** | **CC** | **Cbrm** | **Pia** | **Pons & BS** | **Cblm** | **Other comments** |
| 4009 | ~2.5 | + | ++ | + | - | ++ | NA | + | Found dead (frozen brain). Vascular dilation in pia and cerebellum. |
| 4010 | ~4 | + | ++ | - | - | - | NA | NA | Found dead (frozen brain). |
| 4011 | ~4 | + | ++ | ++ | - | + | NA | - | Widened lateral ventricles. |
| 4012 | ~4 | + | +++ | +++ | - | +++ | NA | +++ | Cell rich areas in lateral ventricle roof and pia, with capillaries, but not the diffuse spread. Widened lateral ventricles.  Glioma-like areas. |
| 4013 | ~4 | + | +++ | ++ | - | ++ | NA | +++ | Glioma-like areas. |
| 4014 | ~4 | + | NA | ++ | + | ++ | NA | ++ | Pial proliferation. |
| 4015 =  Founder #12 | ~4 | + | + | + | + | +++ | + | - | Wide 4^th^ ventricle with cells seeded on the ventricular surface. Hippocampus displays prominent capillaries and increased number of cells. |
| 4025 | ~4 | NA | NA | NA | NA | NA | NA | NA | Found dead (brain not suitable for IHC). |
| 4016 | ~8 | + | + | - | - | + | - | - | Abnormal cell proliferation on the surface of the brain. |
| 4017 | ~8 | + | - | - | - | + | + | - | Marginal increase in cell number in brain stem. |
| 4018 | ~8 | + | ++ | ++ | NA | ++ | NA | +++ | Increased number of cells in hippocampus and increase in capillary vessels. |
| **ID** | **Age (months)** | **Enlarged skulls** | **SVZ** | **CC** | **Cbrm** | **Pia** | **Pons & BS** | **Cblm** | **Other comments** |
| 4019 | ~10 | NA | NA | NA | NA | NA | NA | NA | Found dead (no brain available). |
| 4020 | ~11 | + | ++ | + | - | + | - | ++ | Slightly widened ventricles. |
| 4021 | ~12 | + | + | + | NA | +++ | - | +++ | Widened ventricles. Hippocampus presents with prominent capillaries and an increased amount of cells. |
| 4022 | ~18 | + | NA | ++ | - | + | NA | ++ |  |
| 4023 | ~26 | - | - | - | - | +++ | - | - | Atrophic cerebellum and thin inner granular layer. Also a thin granular layer in hippocampus. Wide 4^th^ ventricle. Small nodules of cells scattered over the surface of the brain. |

SVZ - subventricular zone

CC – corpus callosum

Cbrm – cerebrum

Cblm – cerebellum

BS- brain stem

NA- not available
