# Supplementary material for: Brain Abnormalities and Glioma-Like Lesions in Mice Overexpressing the Long Isoform of PDGF-A in Astrocytic Cells
Source: PLoS One. 2011 Apr 7;6(4):e18303. doi: 10.1371/journal.pone.0018303 (PMC3072383; doi:10.1371/journal.pone.0018303)
Supplement: Table S2 — Frequency (%) of Pdgfr-α, Ki-67, Olig2, Sox2 and Gfap positive cells in individual brains of PDGF-AL transgenic and wt mice. (DOC) [file pone.0018303.s002.doc]

| **Mouse ID** | **Protein** | **Hp** | **SVZ** | **CC** | **TL** | **Cblm** |
| --- | --- | --- | --- | --- | --- | --- |
| #4000  (neoplasia-like)  ~1.5 m | Pdgfr-α  Ki67  Olig2  Sox2  Gfap | 33.2  31.3  67.0  2.3  46.9 | 48.8  23.5  66.2  39  73.2 | 33.2  26.0  60.2  17  27.0 | 39.6  53.3  67.0  33.6  42.3 | 42.8  14  55.8  31.6  32.3 |
| #4008  (increased cellularity)  ~2m | Pdgfr-α  Ki67  Olig2  Sox2  Gfap | 38.1  19  55.3  40.3  50.9 | 34.9  15  51.9  70.3  74.2 | 25.5  2.0  60.2  48.2  44.9 | 25.4  1.2  35.6  43.6  38.7 | 41.2  13  47.7  46.7  65.8 |
| #4  (wt)  ~2m | Pdgfr-α  Ki67  Olig2  Sox2  Gfap | 8.4  1.4  19.5  4.0  51.7 | 7.9  3.3  37.0  0.6  40.0 | 7.9  ND  52.9  1.9  17.3 | 9.8  1.4  25.5  0.6  9.8 | 0.5  0  6.6  13.7  4.1 |

Abbreviations: Hp - hippocampus, SVZ- subventricular zone, CC-corpus callosum, TL- temporal lobe, Cblm –cerebellum.
